# Expression and Purification of a Mammalian P2X7 Receptor from Sf9 Insect Cells

**DOI:** 10.21769/BioProtoc.2544

**Published:** 2017-09-05

**Authors:** Akira Karasawa, Toshimitsu Kawate

**Affiliations:** Department of Molecular Medicine, Cornell University, Ithaca, NY, USA

**Keywords:** P2X7, Sf9, Baculovirus expression system, Eukaryotic membrane protein

## Abstract

The P2X7 receptor is an extracellular ATP-gated ion channel found only in eukaryotes (Bartlett *et al*., 2014). Due to its unique properties among P2X receptors, such as formation of a large conductance pore, the P2X7 receptor has been implicated in devastating diseases like chronic pain (North and Jarvis, 2013). However, mechanisms underlying the P2X7 specific properties remain poorly understood, partly because purification of this eukaryotic membrane protein has been challenging. Here we describe a detailed protocol for expressing and purifying a mammalian P2X7 receptor using an insect cell-baculovirus system. The P2X7 receptor is expressed in Sf9 insect cells as a GFP fusion protein and solubilized with a buffer containing Triton X-100 detergent. The P2X7-GFP fusion protein is then purified in a buffer containing dodecyl maltoside using Strep-Tactin affinity chromatography. Following enzymatic cleavage of the attached GFP and Strep-tag by thrombin, the P2X7 receptor is isolated using size exclusion chromatography. This method typically yields ~2 mg of purified protein from 6 L of Sf9 culture. Purified protein can be stored with a buffer containing 15% glycerol at 4 °C for at least 2 months and used for a variety of functional and structural studies (Karasawa and Kawate, 2016).

## [Background]

The P2X7 receptor is one of the seven subtypes of the purinergic P2X receptor family and has been a promising novel drug target for a wide range of diseases such as neurodegenerative disorders, epilepsy, and neuropathic pain (North and Jarvis, 2013; Bhattacharya and Biber, 2016). Despite the well-documented clinical relevance, mechanisms underlying P2X7 specific functions are unclear. For example, it remains controversial whether P2X7 itself converts into a large pore or if P2X7 activation leads to an opening of another large-conductance channel such as pannexin1 (Alves *et al*., 2014).To unambiguously unravel the P2X7 receptor specific mechanisms, it is desirable to investigate the properties of this membrane channel *in vitro* in the absence of other proteins. It is also extremely advantageous to capture snapshots of the P2X7 receptor conformations throughout its gating cycle using techniques like X-ray crystallography and cryo-electron microscopy. However, purification of eukaryotic membrane proteins is nontrivial due to low expression levels and instability in detergents. Furthermore, complex folding mechanisms and necessary post-translational modifications force researchers to use eukaryotic host cells, which is time and cost consuming. Though several laboratories have established their own protocols for purifying eukaryotic membrane proteins using insect cells (Karakas *et al*., 2011; Hattori and Gouaux, 2012), it is often challenging to mimic experimental conditions simply based on the methods reported in research papers, which normally lack tips and special notes due to space limitation. This protocol aims to provide an in-depth guide for expressing and purifying eukaryotic membrane proteins using an insect cell/baculovirus system. Based on this protocol, we have successfully purified milligram quantities of a mammalian P2X7 receptor, which we used to determine its crystal structures (Karasawa and Kawate, 2016).

### Materials and Reagents

1. Generic pipette tips (VWR, catalog numbers: 613-0741, 613-2133, 613-0746)
2. Petri dish, 100 × 15 mm sterile disposable, polystyrene (VWR, catalog number: 470175-016) *Manufacturer: Akro-Mils/Myers Industries, catalog number: 2900.*
3. Conical bottom 15 ml and 50 ml polypropylene tubes (Greiner Bio One International, catalog numbers: 188280, 227270)
4. Tube, with snap-on cap, polypropylene, 1.5 ml, 11 × 38 mm (Beckman Coulter, catalog number: 357448)
5. Neptune Microcentrifuge Tubes with Attached Flat Caps1.5 ml, 2 ml (Biotix, catalog numbers: 4445.X, 3765.X)
6. Greiner CELLSTAR multi well culture plates 6 wells (TC treated with lid) (Greiner Bio One International, catalog number: 657160)
7. Syringe filter PVDF 0.22 μm 13 mm diameter (CELLTREAT Scientific Products, catalog number: 229742)
8. orning 150 ml vacuum filter/storage bottle system, 0.22 μm, sterile (Corning, catalog number: 431154)
9. micon Ultra-4 Centrifugal Filter Units MWCO 100 kDa (EMD Millipore, catalog number: UFC810024)
10. Superdex 200 increase 10/300 GL (GE Healthcare, catalog number: 28990944)
11. Syringe (BD, catalog numbers: 329464 and 305559)
12. CELLSTAR Serological Pipettes 2, 5, 10, 25, 50 ml (Greiner Bio One International, catalog numbers: 710107, 606107, 607107, 760107, 768180)
13. Sf9 cells (Thermo Fisher Scientific, Gibco™, atalog number: 11496015)
14. pNGFP-FB3 vector (developed in the Kawate lab) harboring a P2X7 receptor gene
  Note: In this protocol, panda P2X7 gene (NCBI Reference Sequence: XP_002913164.2) is used.
15. MAX Efficiency DH5α competent cells (Thermo Fisher Scientific, Invitrogen™, catalog number: 18258012)
16. MAX Efficiency DH10Bac competent cells (Thermo Fisher Scientific, Invitrogen™, catalog number: 10361012)
17. Liquid nitrogen (Airgas, catalog number: NI 180LT230)
18. Nitrogen gas (Airgas, catalog number: NI HP300)
19. Sf-900 III serum free media (Thermo Fisher Scientific, Gibco™, catalog number: 12658027)
20. SOC medium (Quality Biological, catalog number: 340-031-671)
21. E.Z.N.A. Plasmid Mini Kit (Omega Bio-tek, catalog number: D6942-02)
22. Phenol-chloroform (Sigma-Aldrich, catalog number: 77618)
23. Chloroform (Avantor Performance Materials, J.T. Baker^®^, catalog number: 9257-02)
24. 100% ethanol (Decon Labs, catalog number: V1016TP)
25. HyClone penicillin-streptomycin 100× solution (GE Healthcare, HyClone™, catalog number: SV30010)
26. StrepTactin Sepharose High Performance (GE Healthcare, catalog number: 28-9355-99)
27. Human-Thrombin (Haematologic Technologies, catalog number: HCT-0020)
28. Cellfectin II (Thermo Fisher Scientific, Invitrogen™, catalog number: 10362100)
29. FuGENE 6 (Promega, catalog number: E2691)
30. jetPRIME (Polyplus-transfection, catalog number: 114-15)
31. Difco agar granulated (BD, Difco™, catalog number: 214530)
32. Bacto tryptone (BD, Bacto™, catalog number: 211705)
33. Bacto yeast extract (BD, Bacto™, catalog number: 212750)
34. Sodium chloride (NaCl) (Fisher Scientific, catalog number: S641-212)
35. Kanamycin sulfate (Thermo Fisher Scientific, Gibco™, catalog number: 15160054)
36. Gentamicin (Thermo Fisher Scientific, Gibco™, catalog number: 15710064)
37. Tetracycline (Sigma-Aldrich, catalog number: 87128)
38. Bluo-gal (Teknova, catalog number: B1210)
39. Isopropyl β-D-1-thiogalactopyranoside (IPTG) (EMD Millipore, catalog number: 420322)
40. Polyethylenimine (Polysciences, catalog number: 23966-1)
41. Sodium hydroxide (NaOH) (Fisher Scientific, catalog number: BP359-212)
42. Phosphate buffered saline (PBS) (Fisher Scientific, catalog number: BP399-20)
43. Leupeptin hemisulfate salt (Sigma-Aldrich, catalog number: L2884)
44. Aprotinin (Geno Technology, G-Bioscience, catalog number: 786-046)
45. Pepstatin (Enzo Life Sciences, catalog number: ALX-260-085-M100)
46. Phenylmethylsulfonyl fluoride (EMD Millipore, catalog number: 52332-25G)
47. Triton X-100 (Anatrace, catalog number: T1001)
48. Tris-base (VWR, catalog number: 97061-794)
49. EDTA (Sigma-Aldrich, catalog number: EDS)
50. n-Dodecyl-β-D-Maltopyranoside (Anatrace, catalog number: D310)
51. Hydrochloric acid (HCl) (VWR, BDH^®^, catalog number: BDH7204-4)
52. *d*-Desthiobiotin (Sigma-Aldrich, catalog number: D1411)
53. Glycerol (Alfa Aesar, catalog number: A16205)
54. LB-Bac plate (see Recipes)
55. Polyethylenimine (see Recipes)
56. Resuspension buffer (see Recipes)
57. Solubilization buffer (see Recipes)
58. Washing buffer (see Recipes)
59. Elution buffer (see Recipes)
60. SEC buffer (see Recipes)

### Equipment

1 Baker SterilGARDR II Biological safety Cabinet SG-600 (The Baker, model: Baker SterilGARDR II SG 600)
2 Isotemp digital-control water baths (Fisher Scientific, model: Model 2310)
3 Corning 250 ml polycarbonate Erlenmeyer flask with vent cap (Corning, catalog number: 431144)
4 Innova 44 shaker (Eppendorf, New Brunswick™, model: Innova^®^ 44, catalog number: M1282-0000)
5 Hemocytometer (Daigger Scientific, catalog number: EF16034F)
6 Vortex Genie 2 (Scientific Industries, model: Vortex Genie 2, catalog number: SI-0236)
7 Evosf1 microscope (10× objective at 40% gain)
8 1 L bottle with screw-on cap, polypropylene, 97 × 167 mm (Beckman Coulter, catalog number: 355676)
9 JS-4.2 rotor (Beckman Coulter, model: JS-4.2, catalog number: 339080)
10 Bottle assembly, polypropylene, 250 ml, 62 × 120 mm (Beckman Coulter, catalog number: 356011)
11 JA-14 rotor (Beckman Coulter, model: JA-14, catalog number: 339247)
12 Original Pipet-Aid pipette controller (DRUMMOND Scientific, catalog number: 4-000-110)
13 4635 Cell disruption vessel (Parr Instrument, model: 4635 Cell Disruption Vessel)
14 Bottle, assembly, polycarbonate, 70 ml, 38 × 102 mm, 1-1/2 × 4 in, Aluminum Cap (Beckman Coulter, catalog number: 355622)
15 Type 45 Ti rotor (Beckman Coulter, model: Type 45 Ti, catalog number: 339160)
16 Thomas pestle tissue grinder assemblies with serrated pestles 10 ml (Thomas Scientific, catalog number: 3431E15)
17 500 ml plastic beaker
18 NanoDrop 2000 (Thermo Fisher Scientific, Thermo Scientific™, model: NanoDrop™ 2000, catalog number: ND-2000)
19 Centrifuge 5810R (Eppendorf, model: 5810 R, catalog number: 5811000428)
20 Centrifuge 5424 (Eppendorf, model: 5424, catalog number: 022620401)
21 J6-MI High-capacity centrifuge (Beckman Coulter, model: J6-MI, catalog number: 360291)
22 Adaptor (Beckman Coulter, catalog number: 356096)
23 Avanti J-25I floor-model, refrigerated centrifuge (Beckman Coulter, model: Avanti J-25I, catalog number: 363106)
24 Optima LE-80K (Beckman Coulter, model: Optima™ LE-80K, catalog number: 365668)
25 Optima TLX (Beckman Coulter, model: Optima™ TLX, catalog number: 361544)
26 TLA-100.3 Rotor (Beckman Coulter, model: TLA-100.3, catalog number: 349481)
27 Adapter, delrin, tube, 11 mm dia (Beckman Coulter, catalog number: 355919)
28 AKTA purifier (GE Healthcare, model: ÄKTApurifier 10, catalog number: 28406264)
29 Econo-Pac chromatography columns (Bio-Rad Laboratories, catalog number: 7321010EDU)
30 End caps, micro Bio-Spin chromatography columns (Bio-Rad Laboratories, catalog number: 7311660EDU)
31 Mettler Toledo–XP204–Analytical Balance (Mettler-Toledo International, model: XP204S/M, catalog number: 11130054)
32 OHAUS Harvard Trip Balances (OHAUS, catalog number: 80000005)
33 Milli-Q Reference Water Purification System (EMD Millipore, catalog number: Z00QSV0WW)
34 SevenEasy pH meter (Mettler-Toledo International, catalog number: 51302819)
35 Corning 500 ml polycarbonate Erlenmeyer flask with vent cap (Corning, catalog number: 431145)
36 Corning 2 L polycarbonate Erlenmeyer flask with vent cap (Corning, catalog number: 431255)
37 5 × 7 Inch Top PC-420D stirring plate (Corning, catalog number: 6798-420D)
38 Magnetic stir bars
39 Gravity and vacamatic sterilizers Amsco EAGLE SERIES 3000 (AMSCO, catalog number: 213415)
40 Fraction collector Frac-950 (GE Healthcare, model: Frac-950, catalog number: 18608300)
41 Refrigerator (Panasonic Biomedical, model: MPR-1411)
42 Steadystir digital S56 (Fisher Scientific, catalog number: 14-359-756) or an equivalent homogenizer

Note: This product has been discontinued.

43 F-500BAF, Ice maker (Hoshizaki America, model: F-500BAF)
44 Globe Scientific Ice Bucket with Lid (Globe scientific, catalog number: IBB003P)
45 C1000 Touch Thermal cycler (Bio-Rad Laboratories, catalog number: 1851148)
46 EVOS FL Imaging System (Thermo Fisher Scientific, catalog number: AMF4300)
47 EVOS Light Cube, GFP (Thermo Fisher Scientific, catalog number: AMEP4651)

### Software

1. Vector NTI software 11.5 (Thermo Fisher Scientific)

### Procedure

**Figure 1:**
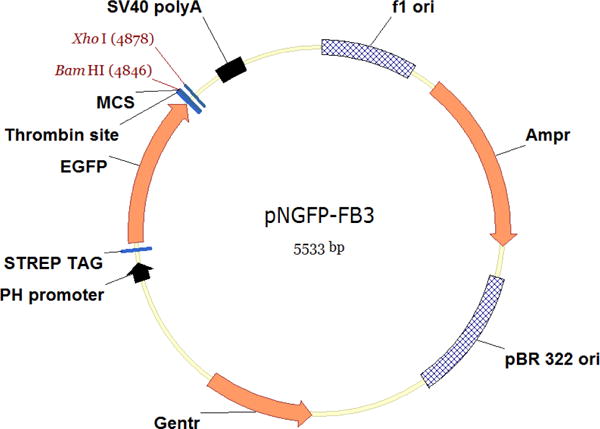
Vector map of pNFGP-FB3 This figure is created using Vector NTI software (Thermo Fisher Scientific).

**Figure 2:**
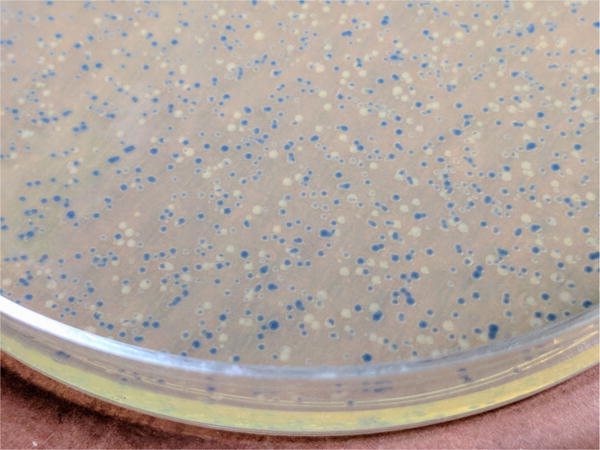
Colonies of DH10Bac transformants The picture was taken 2 days after transformation with the pNFGP-FB3-P2X7 construct.

**Figure 3:**
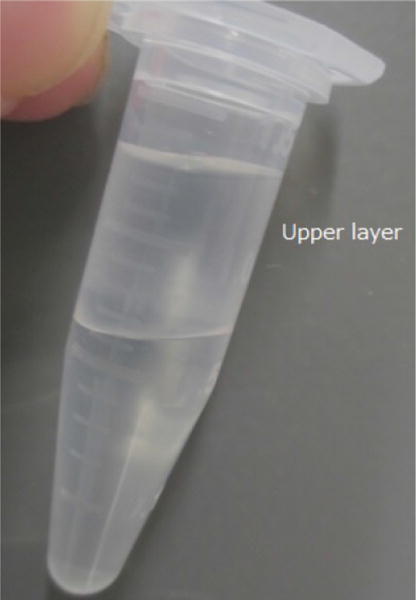
Phase separation after centrifugation at step B14 The upper layer includes bacmids.

**Figure 4:**
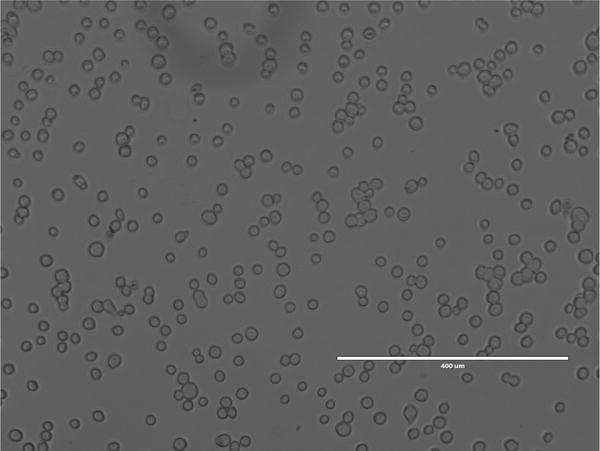
Sf9 cells in 6-well plate before transfection Image was taken using an Evosf1 microscope (10× objective at 40% gain).

**Figure 5:**
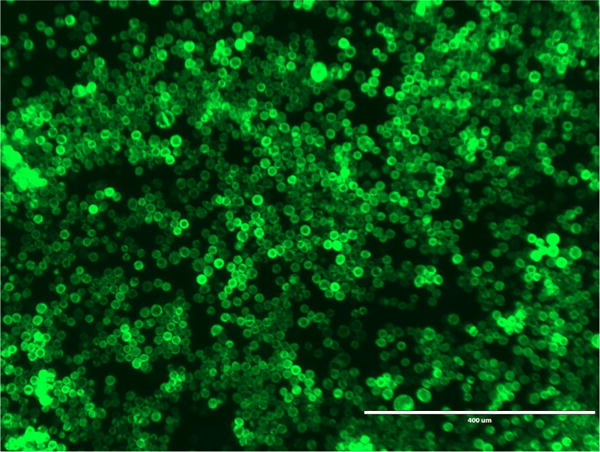
GFP fluorescence from Sf9 cells expressing GFP-P2X7 GFP fluorescence 7 days after transfection. Image was obtained using an Evosf1 microscope (10× objective; 10% gain; Ex: 470/22 Em: 525/50). Scale bar = 400 μm.

**Figure 6:**
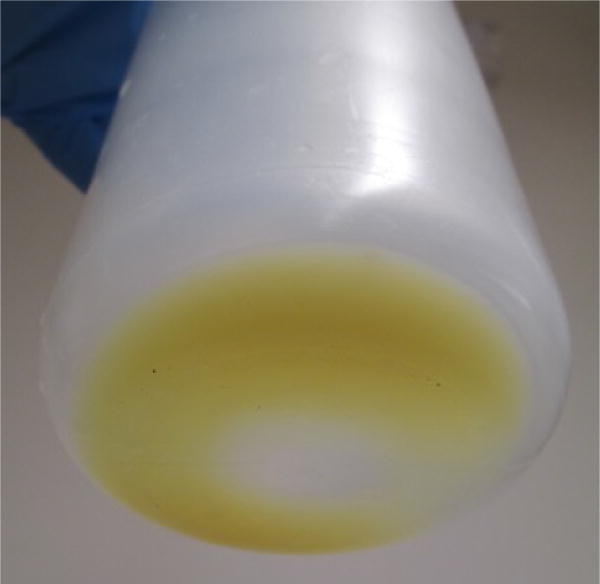
Cell pellet after centrifugation (step E2)

**Figure 7:**
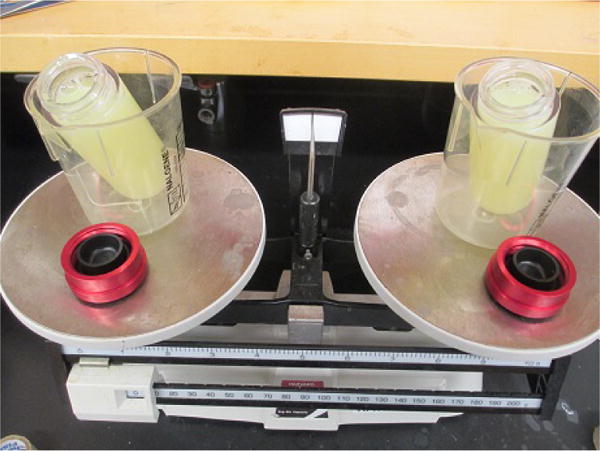
Cell lysates in the ultracentrifuge tubes (step E7) Two tubes need to be balanced precisely.

**Figure 8:**
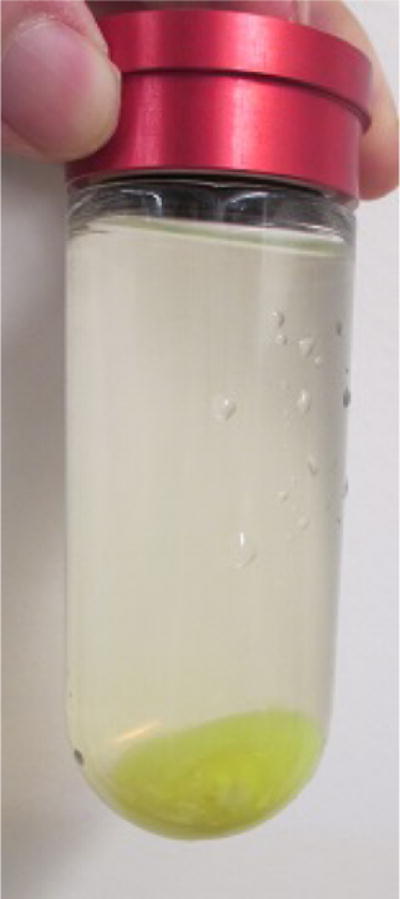
Collected membrane fraction after ultracentrifugation (step E9)

**Figure 9:**
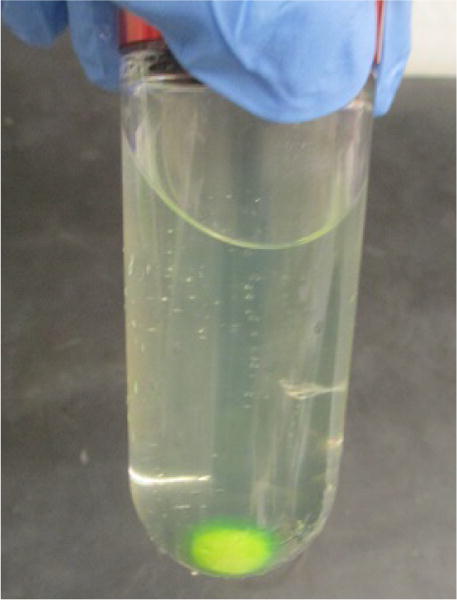
Separated soluble fraction after ultracentrifugation (step E15)

**Figure 10:**
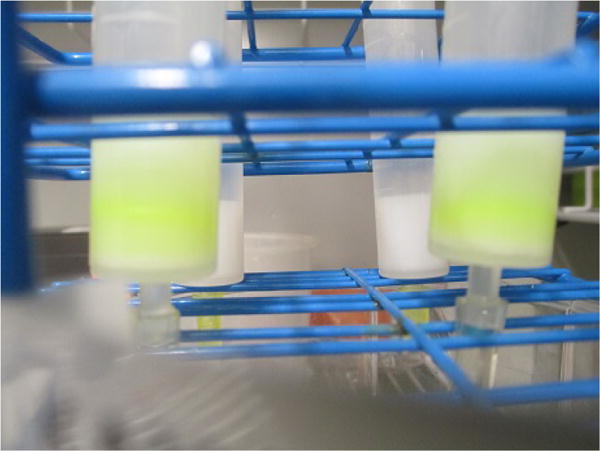
GFP-P2X7 bound to Strep resin

- Sf9 culture (perform in a tissue culture hood except for centrifugation steps)
  1. Sf9 cells in a cryo-vial are stored in liquid nitrogen.
  2. Thaw an aliquot (1–2 ml) of stock Sf9 insect cells (stored in liquid nitrogen) in a 37 °C water bath and resuspend into 10 ml Sf-900 III medium.
  3. Spin down the cells at 125 × *g* for 5 min, remove the medium by pipetting, and gently resuspend the cells in a 15 ml conical tube with 10 ml Sf900 III medium by pipetting up and down.
  4. Transfer the cells into a 250 ml flask and add 15 ml of Sf900 III to make a 25 ml cell suspension.
  5. Incubate the cells in a temperature controlled shaker at 27 °C and at 125 rpm.
  6. Monitor the cell density every day by manually counting the number of cells using a hemocytometer.
  7. When the cell density reaches 3.0–3.5 × 10^6^ cells/ml (normally within 24–48 h), split the cells with Sf-900 III medium to make another 20 ml culture at 0.7 × 10^6^ cells/ml.
  8. Incubate the cells in a temperature controlled shaker at 27 °C and at 125 rpm.
  9. After repeating the steps A6–A8 for 2–3 times, the doubling time should become approximately 24 h. Maintain Sf9 cultures at 0.5–3.5 × 10^6^ cell/ml in a 250 ml flask and grow up in a larger flask for P2 virus infection.
- Preparation of bacmids
  1. The pNGFP-FB3 vector (Figure 1) harbors STREP tag, EGFP, thrombin recognition site, multiple cloning site (MCS) and a stop codon. The P2X7 receptor gene is subcloned between *Bam*HI and *Xho*I sites.
  2. Incubate 25 μl of DH10Bac competent cells with ~100–250 ng of pNGFP-FB3[P2X7] on ice for 30 min.
  3. Heat shock the cells for 45 sec at 42 °C and put them on ice for 2 min.
  4. Add 500 μl of SOC medium into the cells and shake in a temperature controlled incubator at 225 rpm at 37 °C for 4 h.
  5. Harvest the cells by centrifugation at 20,000 × *g* for 1 min and plate them on a LB-Bac plate (see Recipes) (add ~50 μl SOC medium if necessary).
  6. Incubate the LB-Bac plate for 2 days at 37 °C.
  7. Pick up a white colony (Figure 2), inoculate 8 ml of LB-Bac medium, and culture in a temperature controlled incubator at 225 rpm at 37 °C overnight.
  8. Harvest the cells by centrifugation at 2,500 × *g* for 10 min.
  9. Resuspend the cells with 200 μl of ‘Solution 1’ in Plasmid mini kit (Omega Bio-Tek).
  10. Add 200 μl of ‘Solution 2’ in Plasmid mini kit (Omega Bio-Tek) and mix by flipping the tube several times.
  11. Add 270 μl of ‘Solution 3’ in Plasmid mini kit (Omega Bio-Tek) and mix by flipping the tube several times.
  12. Harvest the bacmid containing solution by centrifugation at 20,000 × *g* for 10 min (save supernatant) and place it into a new 1.5 ml tube.
  13. Add 700 μl of phenol-chloroform and mix well by vortexing the tube for a few seconds.
  14. Centrifuge at 20,000 × *g* for 2 min and transfer the upper layer (Figure 3) into a new 1.5 ml tube.
  15. Add 700 μl of chloroform and mix well by vortexing for a few seconds.
  16. Centrifuge at 20,000 × *g* for 2 min and transfer the upper layer into a 2.0 ml tube.
  17. Add 1.4 ml of 100% ethanol and mix well by vortexing for a few seconds.
  18. Chill the tube at −20 °C for 15 min.
  19. Harvest the bacmid by centrifugation at 20,000 × *g* for 15 min at 4 °C.
  20. Rinse the bacmid containing pellet with 1 ml 70% ethanol.
  21. Aspirate the 70% ethanol and dry the bacmid containing pellet in a tissue culture hood for 30 min.
  22. Resuspend the bacmid with 50 μl sterile Milli-Q water in a tissue culture hood. Store bacmids at −20 °C.
- Virus production
  1. Plate Sf9 cells at 0.4 × 10^6^ cells/well into a 6-well tissue culture plate.
  2. Incubate the plate at 27 °C for 1 h (Figure 4).
  3. In the meantime, mix 5 μl of bacmid solution, 10 μl of polyethylenimine solution (see Recipes), and 100 μl of Sf-900 III medium.
  4. Incubate the mixture for 15 min at room temperature and add to the wells of the previously plated 6-well plate by pipetting dropwise.
  5. Incubate the plate at 27 °C for 6–7 days. GFP fluorescence was observed 7 days after transfection using an Evosf1 microscope (10× objective; 10% gain; Ex: 470/22 Em: 525/50) (Figure 5).
  6. Collect the P1 virus containing media and filter sterilize using a 0.22 μm filter. Store the P1 virus at 4 °C.
  7. Inoculate 200 ml of Sf9 cells at 1.0 × 10^6^ cells/ml with 100 μl of P1 virus.
  8. Culture the cells in a temperature controlled incubator at 125 rpm for 3 days at 27 °C.
  9. Collect the P2 virus containing media by centrifugation at 2,500 × *g* for 10 min. Filter sterilize the P2 virus using a 0.22 μm filter and store at 4 °C.
- Expression of P2X7 in Sf9 cells
  1. Set up 1 L Sf9 culture at 0.5 × 10^6^ cells/ml and let them grow for 3 days at 125 rpm at 27 °C.
  2. Split the 1 L culture into 6 L (final density at 0.5 × 10^6^ cells/ml). Add 7.5 ml/L of penicillin-streptomycin and culture in a temperature controlled shaker for 3 days at 125 rpm at 27 °C.
  3. When the cell density reaches 4.0 × 10^6^ cells/ml, infect the Sf9 cells with 30 ml/L P2 virus. Culture at 27 °C for 24 h.
  4. Shift the temperature from 27 °C to 18 °C and culture for another 48 h before harvesting.
- Purification of P2X7 (perform all the steps at 4 °C or on ice)
  1. Transfer the GFP-P2X7 expressing cells into 1 L centrifuge bottles and spin down at 2,000 × *g* for 10 min (JS-4.2 rotor).
  2. After removing the culture media by decanting, resuspend the cells with ~20 ml/bottle of the resuspension buffer (see Recipes). Pellet should be yellow/green (Figure 6). Combine and transfer the cell suspensions into a 250 ml centrifuge bottle.
  3. Harvest the cells by centrifugation at 3,800 × *g* for 10 min (JA-14 rotor).
  4. Carefully remove the resuspension buffer using a pipette and resuspend the cells with 200 ml of a fresh resuspension buffer.
  5. Break the cells by nitrogen cavitation using a cell disruption vessel (600 psi for 20 min; see Videos 1 and 2). Vessel should be incubated at 4 °C after nitrogen loading.
  6. Sediment cell debris by centrifugation at 12,700 × *g* for 10 min (JA-14 rotor).
  7. Transfer the supernatant into three 70 ml ultracentrifuge tubes and balance them using the resuspension buffer. It is critical to balance the centrifuge tubes precisely and to fill it up to the shoulder of the tubes (Figure 7).
  8. Centrifuge at 185,000 × *g* for 1 h (Ti-45 rotor).
  9. Remove the supernatant, resuspend the membrane fraction (pellet, Figure 8) with 2 ml/tube PBS.
  10. Transfer the membrane fraction into a Dounce homogenizer (total volume is 8–12 ml; see Video 3).
  11. Set the homogenizer at ~900 rpm and homogenize the membrane fraction with five strokes going up and down (see Video 4).
  12. Solubilize the homogenized membrane fraction with ~350 ml of the solubilization buffer (see Recipes) by stirring at 300 rpm for 1 h.
  13. Transfer the supernatant into six 70 ml ultracentrifuge tubes and balance them using the solubilization buffer.
  14. Centrifuge at 185,000 × *g* for 1 h (Ti-45 rotor).
  15. Pool the supernatant (Figure 9) into a 500 ml beaker. Add 6 ml of Strep-Tactin resin pre-equilibrated with the solubilization buffer, and stir at 200 rpm for 60 min.
  16. Harvest the resin by centrifugation at 2,500 × *g* for 5 min in a 50 ml tube and transfer into two 25 ml gravity columns.
  17. Wash the resin with 20 ml of the washing buffer (see Recipes) for each column.
  18. Elute the GFP-P2X7 protein with elution buffer (see Recipes; Figure 10).
  19. Concentrate the eluted GFP-P2X7 down to 1 ml using a 100 kDa-cutoff spin column. Measure the protein concentration using a spectrophotometer.
  20. Digest the GFP-P2X7 with thrombin (25:1 [w/w]) overnight. In the meantime, equilibrate a Superdex 200 column with the SEC buffer (see Recipes) using an FPLC.
  21. Remove aggregated protein by centrifugation at 264,360 × *g* for 10min.
  22. Inject the protein into the Superdex 200 column and collect the peak fractions.

### Data analysis

**Figure 11:**
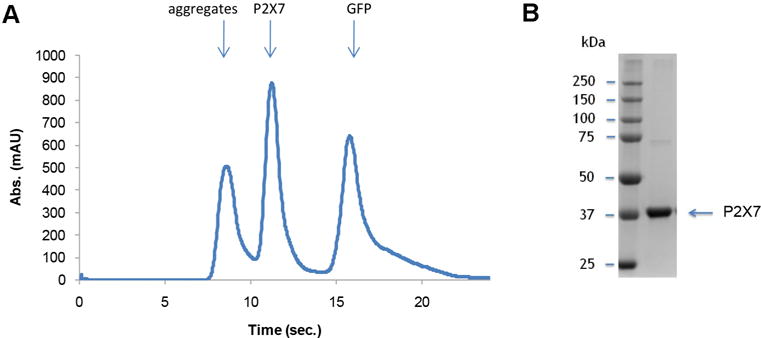
Characterization of purified P2X7 Representative SEC profile (A) and a gel image (B) of purified P2X7.

Quality of the purified P2X7 receptor can be analyzed by SEC and SDS-PAGE (Figure 11). Figure 11A shows a representative SEC profile with a single P2X7 peak, suggesting that the purified P2X7 receptor is monodisperse. A representative SDS-PAGE gel image (Figure 11B) verifies the chemical purity of this sample.

### Notes

1. Even a slightly higher concentration of gentamicin may be too toxic to DH10Bac cells. Use it exactly at 6.7 μg/ml.
2. Cellfectin II (Thermo Fisher Scientific: 10362100), FuGENE 6 (Promega: E2691), and jetPRIME (polyplus, 114–15) could be also used for bacmid transfection.
3. P2 virus should be always prepared fresh. Noticeable reduction of P2X7 receptor expression is observed with the usage of more than one week old virus.
4. Temperature shift from 27 °C to 18 °C increases the expression level of P2X7 by more than four fold.
5. Overgrown cells (> 5.0 × 10^6^ cells/ml) result in low infection. Sf9 cells should be maintained within 30 passages (about 2 months).
6. ESF921 medium (Expression system: 96-001-01) can also be used for Sf9 cell culture.
7. Inclusion of glycerol in both elution and SEC buffers is necessary for avoiding aggregation of P2X7.

### Recipes

1. LB-Bac plate (1 L)
  10 g trypton
  5 g yeast extract
  5 g NaCl
  50 μg/ml kanamycin, 6.7 μg/ml gentamicin, 10 μg/ml tetracycline, 100 μg/ml Bluo-gal, and 40 μg/ml IPTG
  MilliQ H2O to make it 1 L
2. Polyethylenimine
  Dissolve 1 g polyethylenimine in 900 ml MilliQ water
  Adjust the pH to 7.0 with NaOH
  Volume up to 1 L with MilliQ water
  Filter sterilize using a 0.22 μm filter and store at 4 °C
3. Resuspension buffer
  1× PBS
  0.5 μg/ml leupeptin
  2 μg/ml aprotinin
  0.5 μg/ml pepstatin
  0.5 mM PMSF
4. Solubilization buffer
  1× PBS
  2% TritonX-100
5. Washing buffer
  100 mM Tris
  150 mM NaCl
  1 mM EDTA
  0.5 mM Dodecyl-maltoside
  Adjust pH to 8.0 with HCl
6. Elution buffer
  100 mM Tris
  150 mM NaCl
  1 mM EDTA
  0.5 mM Dodecyl-maltoside
  2.5 mM *d*-Desthiobiotin
  15% glycerol
  Adjust pH to 8.0 with HCl
7. SEC buffer
  50 mM Tris
  150 mM NaCl
  15% glycerol
  0.5 mM Dodecyl-maltoside
  Adjust pH to 7.4 with HCl

## Supplementary Material

Video 1

Video 2

Video 3

Video 4

## Figures and Tables

**Video 1:**
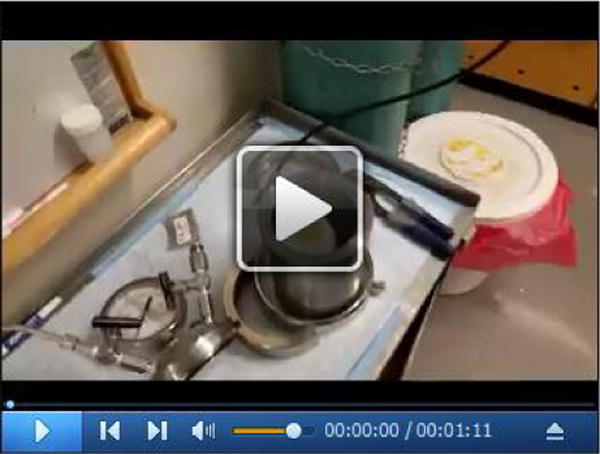
Filling nitrogen gas into the vessel

**Video 2:**
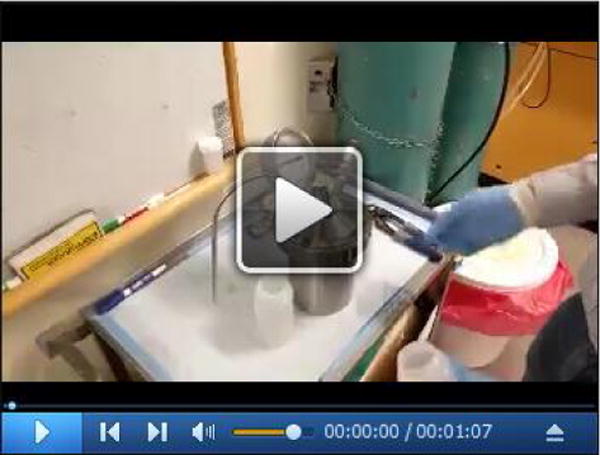
Collecting the lysates after 20 min incubation

**Video 3:**
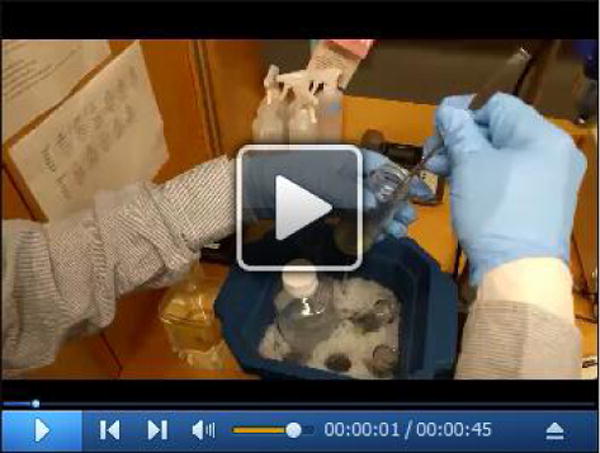
Transfer the membrane fraction into a Dounce homogenizer

**Video 4:**
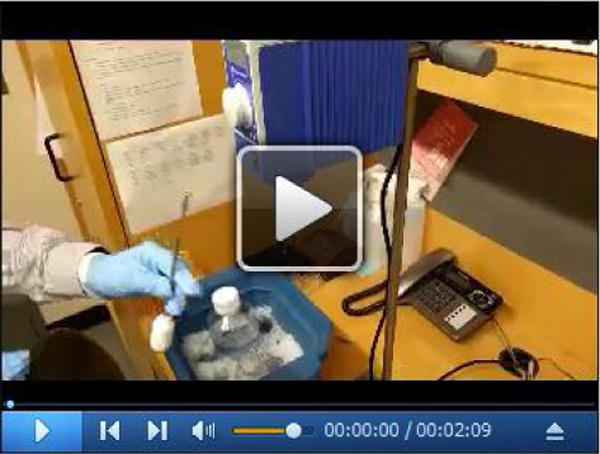
Resuspension of the membrane fraction with a Dounce homogenizer
